# HDX-ESI-MS Reveals Enhanced Conformational Dynamics of the Amyloidogenic Protein β_2_-Microglobulin upon Release from the MHC-1

**DOI:** 10.1016/j.jasms.2008.10.005

**Published:** 2009-02

**Authors:** John P. Hodkinson, Thomas R. Jahn, Sheena E. Radford, Alison E. Ashcroft

**Affiliations:** Astbury Centre for Structural Molecular Biology, Institute of Molecular and Cellular Biology, University of Leeds, Leeds, United Kingdom

## Abstract

The light chain of the major histocompatibility complex class 1 (MHC-1), the protein β_2_-microglobulin (β_2_m), has amyloidogenic properties that arise only upon its dissociation from the MHC-1. Here hydrogen/deuterium exchange electrospray ionization mass spectrometry (HDX-ESI-MS) has been used to compare the solution dynamics of β_2_m in its MHC-1 bound state compared with those of β_2_m as a free monomer. The capability of tandem mass spectrometry to dissociate the MHC-1 into its individual constituents in the gas phase following deuterium incorporation in solution has permitted the direct observation of the exchange properties of MHC-1 bound β_2_m for the first time. The HDX-ESI-MS data show clearly that the H→D exchange of MHC-1 bound β_2_m follows EX2 kinetics and that about 20 protons remain protected from exchange after 17 days. Free from the MHC-1, monomeric β_2_m exhibits significantly different HDX behavior, which encompasses both EX1 and EX2 kinetics. The EX2 kinetics indicate a tenfold increase in the rate of exchange compared with MHC-1 bound β_2_m, with just 10 protons remaining protected from EX2 exchange and therefore exchanging only via the EX1 mechanism. The EX1 kinetics observed for unbound β_2_m are consistent with unfolding of its exchange-protected core with a t_1/2_ of 68 min (pH 7, 37° C). Thus, upon dissociation from the stabilizing influence of the MHC-1, free β_2_m becomes highly dynamic and undergoes unfolding transitions that result in an aggregation-competent protein.

## Graphical Abstract

HDX-ESI-MS reveals that upon dissociation from the MHC-1, the amyloidogenic protein β_2_-microglobulin becomes highly dynamic and undergoes unfolding transitions consistent with aggregation competency.

**Figure:**
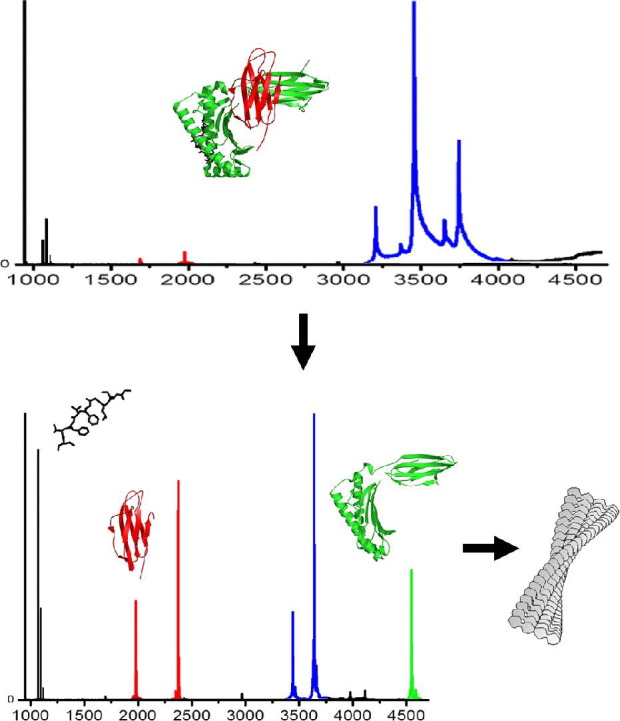

The major histocompatibility complex class 1 (MHC-1), also known as the human leukocyte antigen (HLA), plays an important physiological role in cellular immunity in the majority of vertebrates [1]. MHC-1 molecules, which are expressed on the cell surface, consist of a heavy α chain consisting of three extracellular domains (α1, α2, and α3), to which a light chain, the protein β_2_-microglobulin (β_2_m), is noncovalently bound [2, 3] (Figure 1). The MHC-1 molecules (43.8 kDa) bind small (8- to 10-residue) peptides within a peptide-binding groove that lies between the α1 and α2 domains of the heavy chain [4]. These peptides can be derived from the organism itself or from foreign species such as bacteria or viruses. The role of the MHC-1 is in the differentiation of the origin of the peptides to trigger an immune response against foreign bodies [5, 6, 7, 8].

**Figure 1:**
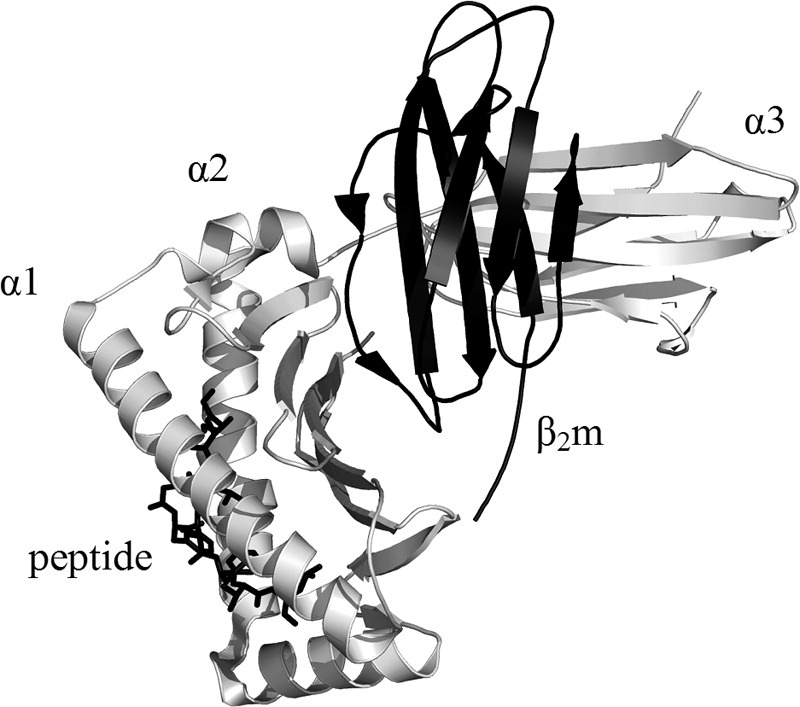
Ribbon diagram of the MHC-1 (PDB 2VLL) [41] showing the heavy chain (α1, α2, α3; gray ribbon), the light chain (β_2_m; black ribbon), and a peptide (black stick) that binds in the cleft formed by the two helical regions (α1 and α2) of the heavy chain. Drawn using PyMOL v.0.99 [42].

The light chain of the MHC-1 (β_2_m) is a 99-residue globular protein that has an immunoglobulin-like fold, a single disulfide bridge, and, in recombinant form, an additional N-terminal methionine residue giving a molecular mass of 11,860.4 Da (Figure 1) [9, 10, 11]. β_2_m is released from the MHC-1 as part of its normal catabolic cycle, whereupon it is degraded by the kidney. However, in renal insufficiency β_2_m is not degraded adequately and the concentration of the protein in the serum increases 25- to 60-fold, with the ultimate result that the protein becomes deposited as insoluble fibrils in the joints [9, 12]. Over the course of years this results in the pathological condition dialysis-related amyloidosis and is characterized by considerable morbidity resulting from joint destruction, pain, and loss of mobility [13, 14].

The structural mechanism of amyloid fibril formation from unbound β_2_m, or indeed from other amyloidogenic proteins, is unresolved and uncertainties remain regarding the identity of the aggregation nucleus and how the fiber elongates, as well as the precise structure of the final fibril itself [15, 16, 17, 18]. The consensus, however, is that for amyloid fibril formation to occur, at least for initially folded, globular proteins, unfolding of the protein from its native state is required [19, 20]. Therefore, an important question to address is: Are there any conformational differences between β_2_m in its nonamyloidogenic, MHC-1 bound state and β_2_m in its assembly-competent, unbound state?

Here we use hydrogen/deuterium exchange monitored by electrospray ionization mass spectrometry (HDX-ESI-MS) [21] to investigate the possibility of rarely populated, unfolded conformations of β_2_m, both when the protein is in its monomeric state and also when it is functioning as the noncovalently bound light chain of the MHC-1 in the presence of a bound peptide. The peptide used in these studies (LLFGYPVYV; 1069.6 Da) is a fragment from human T lymphotropic virus type 1, the causative agent of T-cell leukemia, which is known to bind to the MHC-1 in vivo [22, 23]. HDX-ESI-MS is particularly well suited to uncovering unfolded species within a native protein ensemble because exchange arising from uncorrelated conformational fluctuations (EX2) can be differentiated from exchange arising from transient, large-scale unfolding events (EX1) under appropriate conditions [24, 25]. Furthermore, the ability to use tandem mass spectrometry (MS/MS) to dissociate the MHC-1 in the gas-phase following deuterium incorporation in solution permits the exchange properties of MHC-1 bound β_2_m to be compared directly with those of unbound β_2_m, thus revealing their differing conformational dynamics. Although several publications can be found that discuss the possibility of “hydrogen scrambling” in the gas phase when using MS/MS collision-induced dissociation (CID) to fragment covalent bonds along the amide backbone of a polypeptide (e.g., [26, 27]), previous reports on HDX of noncovalently bound species followed by in-source [28] or MS/MS-induced [29] dissociation have indicated HDX levels of a subunit-specific manner. Thus, in this study we demonstrate that the conformational flexibility of MHC-1 bound β_2_m is significantly restricted, whereas upon dissociation from the stabilizing influence of the MHC-1 heavy chain, free β_2_m has increased conformational dynamics that result in the transient population of potential aggregation-competent conformers within the protein ensemble.

## Experimental

### Mutagenesis and Protein Purification

Protein purification of monomeric β_2_m were carried out as described previously [20]. Preparation of the MHC-1 was carried out according to the published procedure [30].

### Reagents and Solvents

Reagents (ammonium acetate, ammonium formate, deuterium oxide, Tris) and calibrants (horse heart myoglobin and cesium iodide) were obtained from Sigma–Aldrich (Dorset, UK). Solvents were purchased from Fisher Scientific (Loughborough, UK).

### H→D Exchange of the MHC-1 Monitored by ESI-MS and ESI-MS/MS

A stock solution of MHC-1 (150 μM; 1:1:1 M ratio of heavy chain, β_2_m, and peptide, confirmed by ESI-MS) was stored at −80 °C in 10 mM ammonium formate:10 mM ammonium acetate at pH 7.0. This solution was diluted 25-fold into 10 mM ammonium formate:10 mM ammonium acetate in D_2_O [pH 7.0 (corrected) i.e., corrected for deuterium by subtracting 0.4 from the pH reading: pD 7.4; 37 °C] to initiate H→D exchange and then incubated in a water bath (37 °C). Aliquots (70 μL) were removed at selected time points for analysis by positive ESI-MS using a Synapt HDMS mass spectrometer (Waters UK Ltd., Manchester, UK). The samples were infused (20 μL min^−1^) with a syringe pump (Harvard Apparatus, Holliston, MA, USA) and the following instrument parameters were set: capillary voltage 3.5 kV; sample cone 70 V; source temperature 60 °C; trap collision energy 6 V. Mass accuracy was ensured by calibration with a separate introduction of cesium iodide. Before each analysis, 100 μL of buffer was infused through the sample inlet system to saturate the enclosed source with deuterium. For MS/MS CID the leading edge of the most intense MHC-1 ions was selected, followed by dissociation using a trap collision energy of 80 V and argon collision gas. The centered +5 and +6 charge state β_2_m monomer ions were used to calculate the level of deuterium incorporation at each time point. After about 17 days, degradation of the sample rendered further analysis impractical.

### Deuteration of Monomeric Wild-Type β_2_m

Lyophilized β_2_m was dissolved in 99.9% D_2_O (final protein concentration 600 μM) buffered to pH 8.0 (corrected) using 5 mM Tris and incubated at 37 °C for 24 h. Complete protein deuteration was confirmed by positive ESI-MS at pH 7.0 (corrected) in 99.9% D_2_O, buffered by 10 mM ammonium formate:10 mM ammonium acetate, using a Platform II mass spectrometer (Micromass UK Ltd., Manchester, UK). Samples were infused (20 μL min^−1^) with a syringe pump (Harvard Apparatus) and the following instrument parameters were set: capillary voltage 3.5 kV; sample cone 40 V. Mass accuracy was ensured by calibration with a separate introduction of horse heart myoglobin. A mass of 12,051.2 Da was measured for β_2_m (calculated fully deuterated mass 12,051.6 Da). The protein solution was sterilized by syringe filtration through a 0.2-μm Anotop 10 filter (Whatman, Maidstone, UK) and the concentration determined by spectrophotometry at 280 nm (Ultrospec 2100 pro, Amersham Biosciences, Uppsala, Sweden) using an extinction coefficient of 20,065 M^−1^ cm^−1^ [31]. The solution was diluted to a final protein concentration of 500 μM, flash frozen, and stored at −20 °C.

### D→H Exchange of Monomeric Wild-Type β_2_m Monitored by ESI-MS

The deuterated protein solution was thawed on ice and diluted 25-fold into 10 mM ammonium formate:10 mM ammonium acetate at pH 7.0, 37 °C to initiate D→H exchange. The solution was incubated in a water bath (37 °C), from which 8-μL aliquots were removed at selected time points for analysis by positive nanoESI-MS using either an LCT Premier (Waters UK Ltd.) or a Synapt HDMS (Waters UK Ltd.) equipped with a NanoMate (Advion Biosciences, Ithaca, NY, USA) nanoESI interface and sample delivery system. A capillary voltage of 1.8 kV and a nitrogen nebulizing gas pressure of 0.8 psi were set, together with the following instrument parameters for the LCT Premier: sample cone 70 V; ion guide 1 70 V; aperture 1 10 V; ion energy 100 V, or for the Synapt HDMS: sample cone 70 V; source temperature 60 °C; trap collision energy 6 V. Mass accuracy was ensured by calibration with a separate introduction of horse heart myoglobin. This experiment was performed several times on both the LCT Premier and the Synapt HDMS, giving identical results within experimental error (i.e., 67.6 ± 3 min).

### H→D Exchange of Monomeric Wild-Type β_2_m Monitored by ESI-MS

β_2_m (500 μM) in 5 mM Tris buffer was diluted 25-fold into 99.9% D_2_O buffered to pH 7.0 (corrected) with 10 mM ammonium formate:10 mM ammonium acetate at 37 °C to initiate H→D exchange. The solution was incubated in a water bath (37 °C). Aliquots (30 μL) were removed at selected time points for analysis by positive ESI-MS using an LCT Premier (Waters UK Ltd.) mass spectrometer. Samples were infused (20 μL min^−1^) with a syringe pump (Harvard Apparatus) and the following instrument parameters were set: capillary voltage 1.8 kV; sample cone 70 V; ion guide 1 70 V; aperture 1 10 V; ion energy 100 V. Before each analysis, 100 μL of buffer was infused through the sample inlet system to saturate the enclosed source with deuterium. The instrument was calibrated with a separate introduction of horse heart myoglobin.

### Analysis of the Mass Spectrometry Data

The raw data were processed using MassLynx v.4.1 software (Waters UK Ltd.). The data were smoothed (twice using the Savitsky–Golay algorithm) and centered (peak area).

To establish the contribution of the overlapping sodium and potassium salt adducts of the EX1 peaks to the EX2 peaks, each spectrum was fitted to two sets of Gaussian curves centered on the EX1 and EX2 base peaks using Igor Pro v.5.4 software (Wavemetrics Inc., Portland, OR, USA). For the nanoESI-MS analyses, the base peaks were linked to three additional peaks: base peak −28 Da, base peak +22 Da (Na^+^), and base peak +38 Da (K^+^). These peak positions were determined in each spectrum by linking them and forcing them to move with their respective base peak, whereas their area in each spectrum was determined by their ratio relative to the base peak, observed in the initial and final spectra and assumed to be constant in their relative intensity to the base peak throughout exchange. For ESI-MS analyses, each base peak was linked to two additional adduct peaks; base peak +44 Da (2Na^+^) and base peak +60 Da (Na^+^ + K^+^), which required additional Gaussian curves to adequately describe the data. The accurate area and mass of each peak were determined using this method and these data were used to plot the peak mass versus time course and also the areas of the individual EX1 and EX2 peaks relative to the area of the sum of the peaks versus time.

## Results and Discussion

### HDX-MS and HDX-MS/MS of Intact MHC-1

To characterize the behavior of the amyloidogenic protein β_2_m when in its bound state as the light chain of the MHC-1, HDX was carried out on the intact, noncovalently bound MHC-1 by dilution of a protic solution into a 25-fold volume excess of D_2_O at pH 7 (corrected), 37 °C. H→D, rather than D→H, exchange was chosen as the more appropriate method for studying the intact complex because of the inherent difficulty in fully deuterating a highly protected, noncovalently bound complex. The progress of HDX was monitored by ESI-MS analysis of reaction mixture aliquots taken at selected time points. Standard ESI-MS was used for the H→D continuous labeling experiments because the closed nature of such atmospheric pressure ion sources averts any D→H back exchange during the ionization process. Slightly higher flow rates than normal were used to minimize exchange during the analysis (i.e., flow rates of ∼20 μL min^−1^ rather than 5–10 μL min^−1^). Using the more open-style nanoESI (i.e., flow rates of <100 nL min^−1^) source housing designs, back exchange of the rapidly exchanging, labile protons was found to occur during the ionization process.

**Figure 2:**
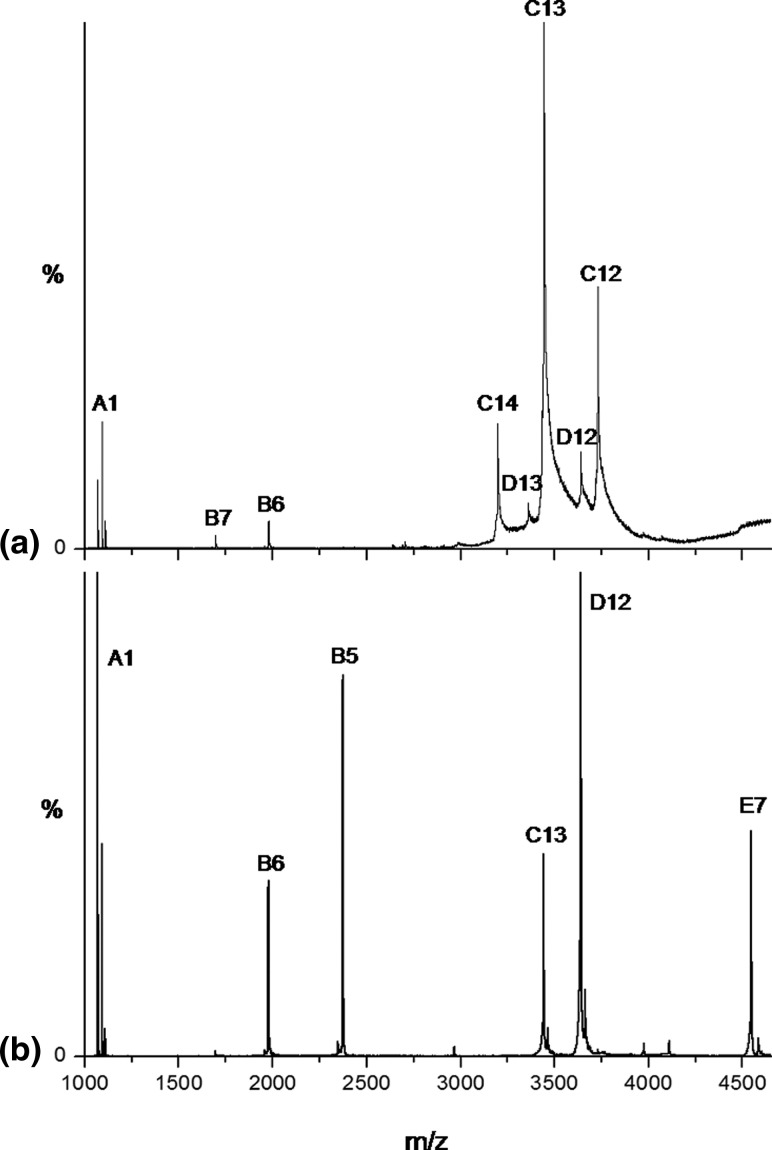
(**a**) ESI-MS *m/z* spectrum of the MHC-1 (pH 7.0), showing predominantly intact MHC-1 (C), together with traces of MHC-1 minus peptide (D), free β_2_m (B), and peptide (A). (**b**) ESI-MS/MS *m/z* spectrum showing dissociation of intact MHC-1 (C; +13 charge state ions, *m/z* 3442) to yield: MHC-1 minus peptide (D), MHC-1 heavy chain (E), free β_2_m (B), and peptide (A). The number immediately following the letters A, B, C, D, and E indicates the charge state of those ions.

The *m/z* spectrum of the MHC-1 (Figure 2a) shows predominantly ions consistent with the intact MHC-1, i.e., +12, +13, and +14 charge state ions at *m/z* 3729, 3442, and 3196, respectively (component C; measured mass 44,738 Da), accompanied by trace amounts of the free peptide (component A; MH^+^, *m/z* 1071), the free light chain (β_2_m; component B; +6 and +7 charge states, *m/z* 1978 and 1695, respectively; measured mass 11,860 Da), and the MHC-1 minus the peptide (component D; +12 and +13 charge states, *m/z* 3640 and 3360, respectively; measured mass 43,666 Da). To gain specific insights into the HDX properties of β_2_m exclusively when in its bound state within the MHC-1, MS/MS was used to dissociate the intact MHC-1 complex into its individual components and thus present the opportunity to monitor the HDX properties of each protein component individually. Thus the most intense MHC-1 ions, the +13 charge state ions (*m/z* 3442), were selected and fragmented using argon collision gas. The MS/MS spectrum (Figure 2b) indicates the presence of residual trace amounts of the parent ions of the intact MHC-1 (component C; +13 ions, *m/z* 3442), the MHC-1 minus the peptide (component D; +12 charge state ions, *m/z* 3640), the free heavy chain (component E; +7 charge state ions, *m/z* 4545), free β_2_m (component B; +5 and +6 charge state ions, *m/z* 2373 and 1978, respectively), and the free peptide (component A; MH^+^ and MNa^+^ ions, *m/z* 1071 and 1093, respectively). Because all the species detected from the MS/MS analysis originate directly from the intact MHC-1, we can be sure that only HDX labeling of the complex, and not of the minor components seen in the MS spectrum (Figure 2a), is being monitored using this methodology. Thus, any unbound light chain or heavy chain molecules existing in solution would not be *m/z* selected for MS/MS in this experiment.

**Figure 3:**
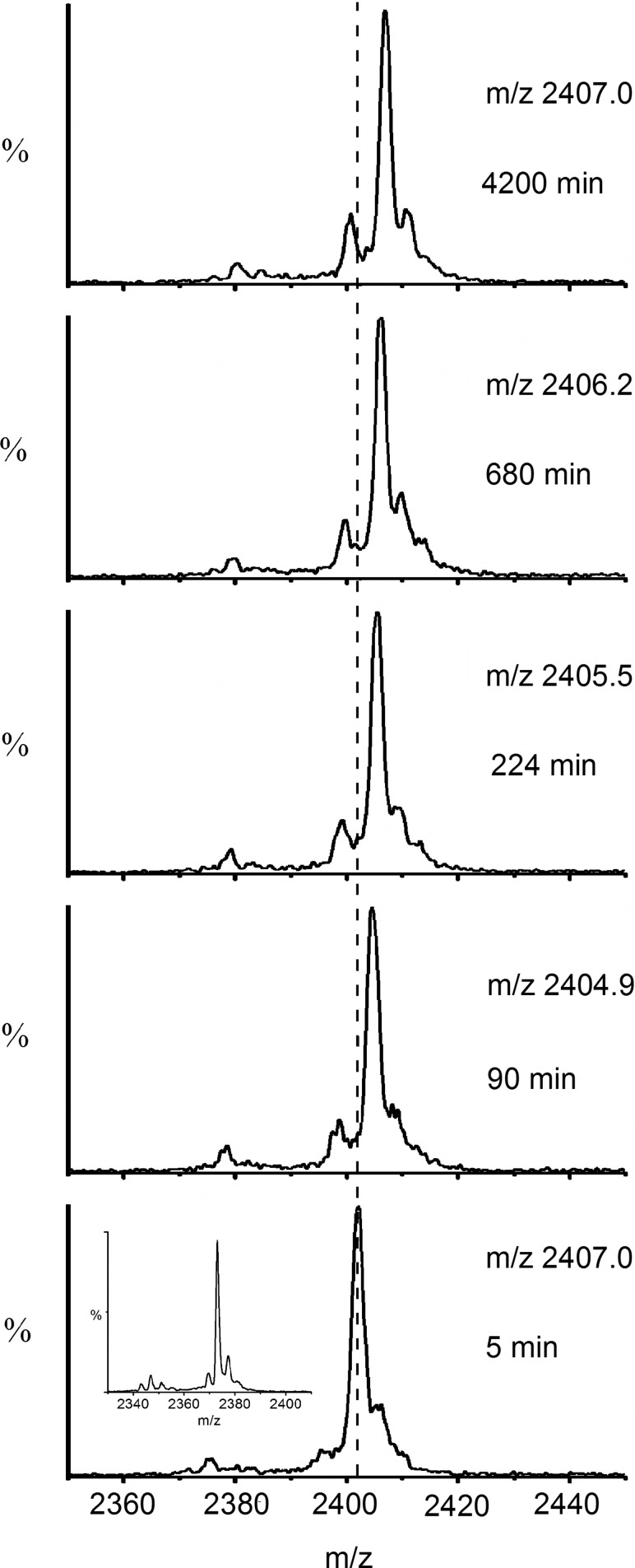
Time course showing H→D exchange (pH 7.0 corrected, 37 °C) of β_2_m, which has been dissociated from intact MHC-1 after HDX by ESI-MS/MS. The +5 charge state ions are highlighted showing *m/z* versus intensity over the time course. The centroided *m/z* values of the ions are indicated on each spectrum and the centroided *m/z* of the first time point is shown throughout as a dashed line. The unexchanged +5 charge state ions (t = 0) are shown in the inset.

**Figure 4:**
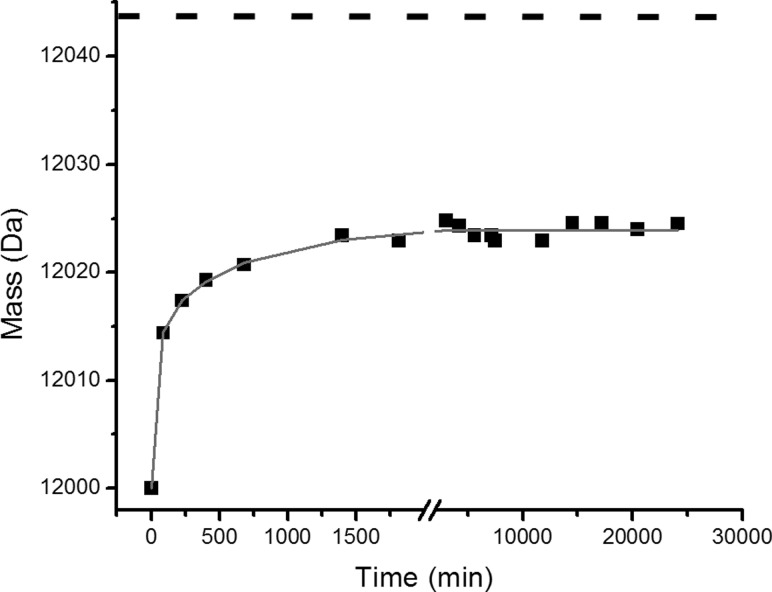
The increase in molecular mass of β_2_m observed by ESI-MS/MS dissociation of the intact MHC-1 over the course of H→D exchange (pH 7.0 corrected, 37 °C). A single-exponential fit (gray line) indicates a rate constant of 0.002 ± 0.0004 min^−1^. The theoretical end point of the reaction (12,044 Da; dashed line) is shown; about 20 protons remain protected.

Focusing on the +5 charge state ions of β_2_m in the MS/MS spectrum (*m/z* 2360–2440) enabled the extent of deuterium labeling during the time course to be assessed (Figure 3). From these data the reaction profile could be formulated, showing the mass increase attributed to deuterium incorporation over time. From this analysis, the exchange of MHC-1 bound β_2_m was found to occur by EX2 kinetics with a rate of 0.002 ± 0.0004 min^−1^ and the theoretical end point of the exchange (12,044 Da) was not reached even after 17 days (Figure 4). Because the analysis of fully deuterated β_2_m using standard ESI-MS has shown that back exchange does not occur during the ionization process—and thus cannot be the reason for the incomplete deuteration observed in this exchange reaction—these results indicate a highly protected core of about 20 protons for which stable hydrogen bonding and/or exclusion of solvent precludes exchange under the conditions used.

### HDX-MS of Unbound β_2_m

Having ascertained the HDX exchange profile of β_2_m when bound within the MHC-1, the conformational characteristics of this protein in its unbound, amyloid-competent state were next investigated to compare the dynamics of β_2_m in its MHC-1 bound and free states. Previous studies have shown that unbound β_2_m retains the native immunoglobulin fold of its bound counterpart [10, 11, 32] and denatures apparently cooperatively when subjected to urea titration [20]. Prior deuteration of monomeric β_2_m (see Experimental section) allows D→H HDX-MS to be performed, whereas for undeuterated protein H→D exchange can be monitored, the two modes generating equivalent but complementary data. For D→H exchange, unbound β_2_m was fully deuterated by dissolution in D_2_O at pH 8 (corrected; see Experimental section) until ESI-MS analysis indicated the maximum mass increase of 190 to 12,051.2 Da. Dilution into a 25-fold volume excess of protonated buffer was then carried out to initiate the exchange reaction (pH 7, 37 °C). Within 2 min nanoESI-MS indicated that 145 out of the possible 190 deuterons had undergone exchange; the remaining 45 deuterons (i.e., those that are highly protected) exchanged more slowly, presumably because they are involved in hydrogen bonding in buried secondary structural elements. An advantage of D→H exchange is that nanoESI-MS can be used to monitor the reaction because all of the extremely labile deuterons exchange in solution within seconds, whereas the slower-exchanging deuterons are unlikely to exchange during the short time in the open, nanoESI source. Further advantages of nanoESI-MS over ESI-MS are that a lower degree of salt adduct ion formation is observed and also less sample is consumed.

**Figure 5:**
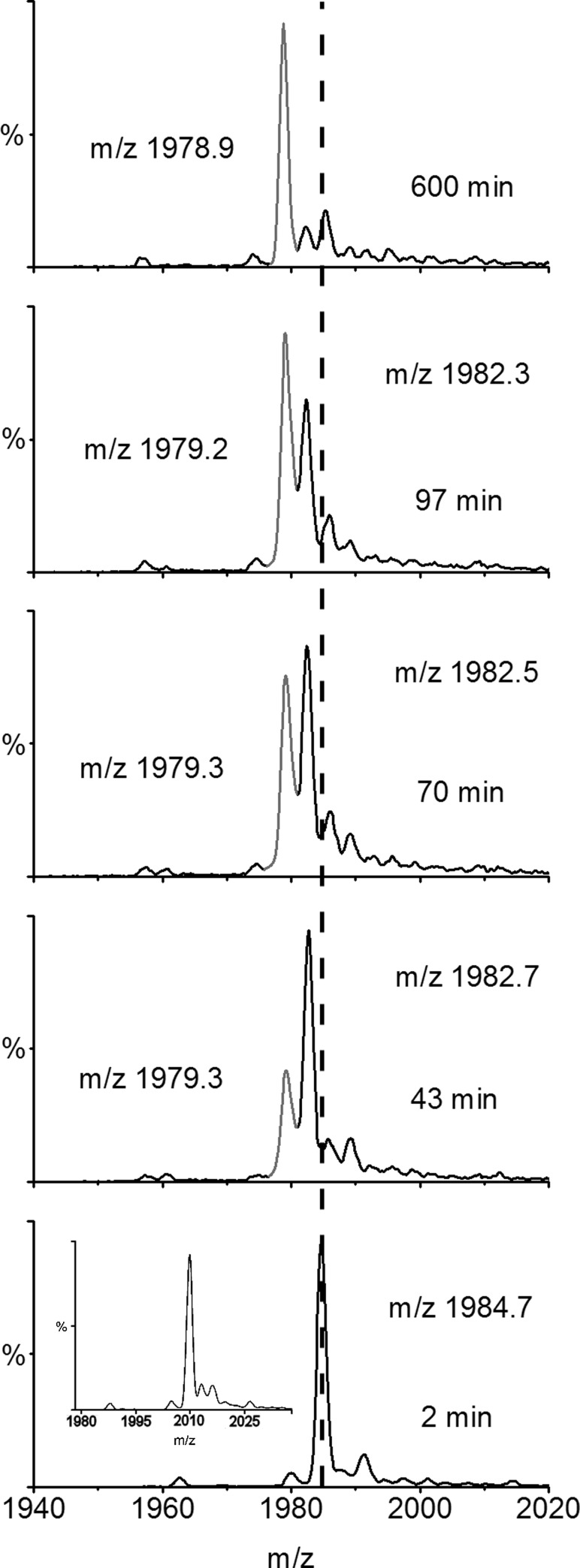
HDX-ESI-MS of unbound, monomeric β_2_m (pH 7, 37 °C) highlighting the +6 charge state ions showing *m/z* versus intensity over the time course of the D→H exchange reaction. The black (higher *m/z*) peaks show the population of protein molecules in solution that are undergoing exchange by an EX2 mechanism alone, whereas the gray (lower *m/z*) peaks indicate the molecules in solution that have experienced exchange by the EX1 mechanism. The centroided *m/z* values of the ions are indicated on each spectrum and the centroided *m/z* of the first time point is shown throughout as a dashed line. The unexchanged +6 charge state ions (t = 0) are shown in the inset.

Figure 5 shows the nanoESI-MS *m/z* spectra highlighting the +6 charge state ions of β_2_m (*m/z* 1940–2020) from the first D→H HDX time point over the following 10 h. A bimodal distribution of peaks is clearly observed (black and gray peaks), as well as a gradual decrease in mass of each peak over time, indicating that exchange is occurring by mixed EX1 and EX2 kinetics. The series of peaks at higher *m/z* (colored black in Figure 5) indicates exchange by an EX2 mechanism whereby deuterons are replaced gradually by protons as a result of small, noncorrelated fluctuations that expose deuterium atoms to solvent, consistent with the conformational dynamics expected for a folded protein. The appearance and subsequent increase in intensity of the series of peaks at lower *m/z* (colored gray in Figure 5) are indicative of EX1 kinetics. Surprisingly for such a stable protein at neutral pH, EX1 exchange indicates a significant unfolding event involving the coordinated exposure of deuterons to solvent. These peaks increase in intensity during the exchange time course, as more and more molecules visit an unfolded, exchange-competent conformation in solution.

**Figure 6:**
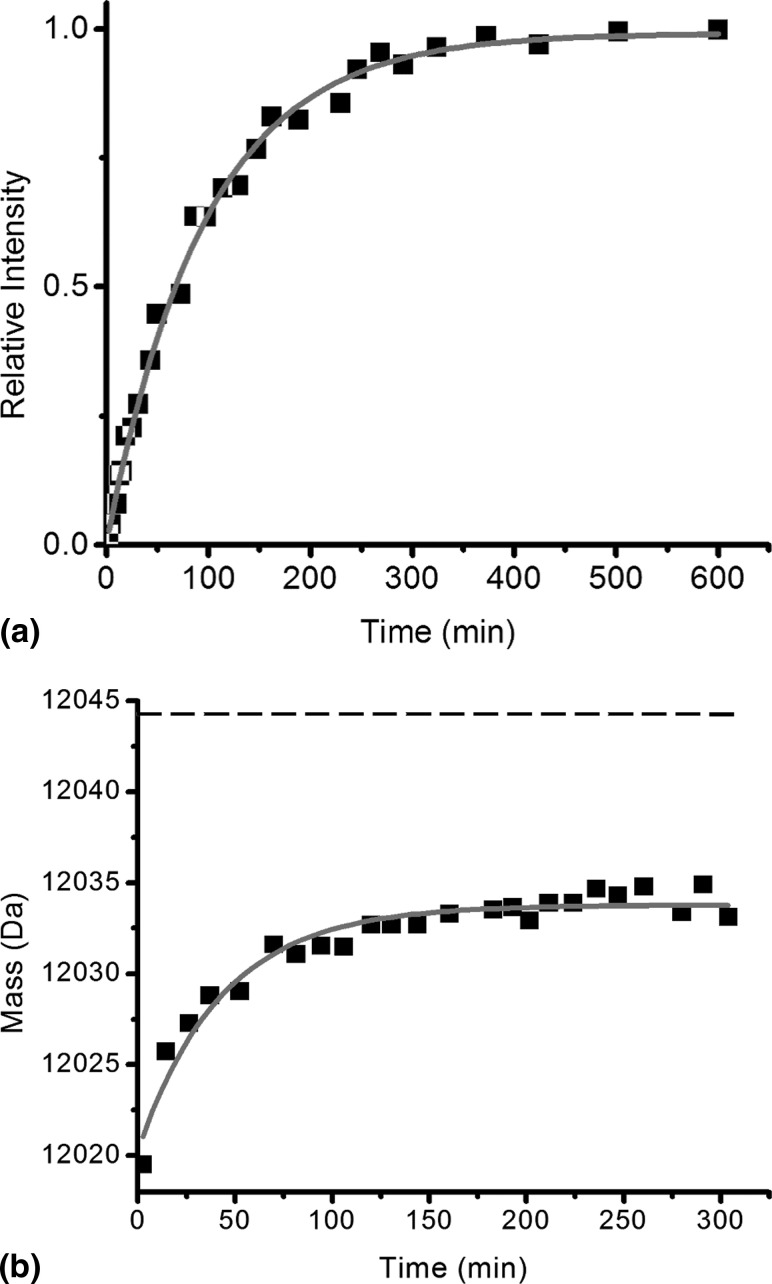
(**a**) The intensity of the EX1 peak relative to the total protein signal during the D→H exchange reaction monitored by HDX-ESI-MS of unbound β_2_m (pH 7, 37 °C). The data fit to a single exponential (gray line) with t_1/2_ = 67.6 ± 3 min. (**b**) The increase in mass of unbound β_2_m undergoing HDX by an EX2 mechanism observed by ESI-MS over the course of H→D exchange (pH 7.0 corrected, 37 °C). A single-exponential fit (gray) indicates a rate of 0.023 ± 0.002 min^−1^. The theoretical end point of the reaction (12,044 Da; dashed line) is shown; about 10 protons exchange only via an EX1 mechanism.

To determine the frequency with which the protein molecules undergo the unfolding event that gives rise to EX1 kinetics, and also the extent of that unfolding event, the mass and intensity of the two series of peaks require accurate evaluation. This presents a challenge because the two series of peaks lie in close proximity on the *m/z* scale, especially during the later time points of the exchange reaction when the lower mass peaks (representing molecules that have undergone EX1 exchange and referred to here as the EX1 peak) become more intense and the low levels of sodium and potassium adduct ions associated with these peaks overlap with the higher mass peaks representing protonated/deuterated molecules that have not undergone EX1 exchange (referred to here as the EX2 peak). An in-house Gaussian-fitting procedure was developed that circumvented this problem for the EX1-related peaks by taking into account the relative intensities of the salt adducts (see Experimental section). Thus, Figure 6a shows the intensity of the EX1-related peak relative to the sum of both EX1 and EX2 related peaks versus time, from which t_1/2_ is estimated as 67.6 ± 3 min.

To enable an accurate analysis of the rate of exchange for the EX2 peak, the exchange was repeated in H→D mode, whereby the increase in mass during the exchange process avoids the interference of the sodium and potassium adduct ions of the EX1 peak with the now lower *m/z* EX2 peak. Standard ESI-MS was used in this case to avoid back exchange during the ionization process. The mass of the EX2 peak was thus measured accurately throughout the time course (Figure 6b). The EX2 rate of exchange from these data was calculated to be 0.023 ± 0.002 min^−1^, some ten times faster than the EX2 rate of HDX observed for β_2_m when bound as the light chain of the MHC-1. The calculated mass of the final, deuterated protein under these solution conditions (i.e., 4% protonated solvent, 96% deuterated solvent) is 12,043.6 Da (183 Da higher than the protonated protein). In contrast, the measured mass of the EX2 peak reached a maximum of 12,033.8 Da at 100 min, indicating a protected core of about 10 protons within the protein's native structure that exchange exclusively via the EX1 mechanism. NMR studies undertaken under these conditions identified nearly 25 slow-exchanging residues located in the B, C, E, and F β-sheet strands, i.e., clustered around the disulfide bridge connecting strands B and F (data not shown), which is consistent with published NMR data [33]. Among these approximately 25 residues are the 10 protons observed here to exchange exclusively via an EX1 mechanism. Although the NMR data provide residue-specific information, they do not distinguish between EX1 and EX2 exchange mechanisms. Thus the HDX-ESI-MS and HDX-NMR data are completely complementary.

These HDX-ESI-MS data show clearly that when β_2_m is bound in the MHC-1, its H→D exchange follows EX2 kinetics with a rate of exchange of 0.002 ± 0.0004 min^−1^, and indicates that approximately 20 protons are highly protected from exchange. In comparison, when in an unbound state free from the MHC-1, β_2_m exhibits very different characteristics exemplified by HDX mechanisms that encompass both EX1 and EX2 kinetics. The EX2 kinetics show a tenfold increase in the rate of exchange (0.023 ± 0.002 min^−1^) compared with MHC-1 bound β_2_m, and approximately 10 highly protected protons exchange exclusively via an EX1 mechanism rather than by combined EX1/EX2 kinetics. The EX1 data observed for unbound β_2_m are consistent with a substantial cooperative unfolding event. Thus, binding to the MHC-1 results in a significant damping of the conformational dynamics of β_2_m, consistent with stable, macromolecular, protein complex architecture.

## Conclusions

ESI-MS is a powerful technique with which to monitor protein conformational dynamics using HDX. One of its key features is the ability to follow multiple exchange mechanisms simultaneously, as exemplified here by the observation of concurrent EX1 and EX2 kinetics in the case of unbound β_2_m. Thus, conformational changes undergone by only a fraction of the protein molecules can be observed and useful structural information gained. Other, perhaps more evident, features are the ability to monitor protein mass changes to within 0.01% accuracy, thus giving precise information regarding the extent of HDX having taken place, and also the capability of real-time reaction monitoring to generate immediate results consuming only microliter volumes of solutes. Furthermore, ESI-MS/MS permits surveillance of the solution dynamics of individual components within large noncovalently bound complexes, which is an important and unique attribute of the technique. Although HDX-MS of unbound β_2_m, as a mixture with its oxidized C-terminal methionine analogue, has previously been shown to follow mixed EX1/EX2 mechanisms [34, 35], here HDX-ESI-MS/MS has enabled a direct comparison to be made for the first time between the conformational dynamics of MHC-1 bound and free β_2_m. To detect MHC-1 bound β_2_m, the exclusive selection of MHC-1 ions by the first analyzer after HDX, and the subsequent fragmentation of these ions to release β_2_m for *m/z* analysis by the second analyzer, ensured that only bound protein was examined in these experiments.

The comparison between MHC-1 bound and unbound β_2_m highlights a remarkable change in the conformational dynamics of β_2_m on its release from the MHC-1. These observations are of significance, given that partial or more complete unfolding is considered to be the key initiating step in protein aggregation processes that lead to disease-related fibril formation [19, 36, 37]. The binding interface between the light and heavy chains of the MHC-1 has been probed by X-ray crystallography, the data revealing a large (1348 Å^2^) interface involving hydrogen bonding, salt bridges, and hydrophobic interactions that stabilize the complex [3, 32, 38]. The larger number of protected residues within β_2_m when MHC-1 bound observed here is consistent with these additional interactions and associated increase in stability upon complex formation, as well as with the reduction in the solvent accessible surface area (30%) [32, 38].

The EX1 process observed for HDX of unbound β_2_m does not lead directly to complete HDX of the protein, in that further exchange is seen to occur following EX1 exchange via an EX2 mechanism. The options for this phenomenon are either that it represents subglobal unfolding or that the rate of the refolding event is such that insufficient time is available for all labile protons to undergo exchange [39]. Because there is evidence that β_2_m retains some residual structure even in the acid-unfolded state at pH 2.5 [38, 40], some residual structure in the unfolded state at pH 7 may well be expected, especially given the presence of the disulfide bond (Cys25—Cys80) connecting β-strands B and F, which remains intact in these experiments.

The stabilizing effect of the noncovalently bound MHC-1 macromolecular structure on the conformational dynamics of β_2_m provides a clue for therapeutic intervention against dialysis-related amyloidosis. Thus binding of small molecules, aptamers, or antibodies could be used to stabilize the native fold of β_2_m in a similar manner and, consequently, prohibit the conformational flexibility necessary to trigger amyloidosis. The HDX-ESI-MS methods developed here represent an efficient and practical assay to test the efficacy of such inhibitors by monitoring their ability to affect the conformational dynamics of unbound, amyloidogenic β_2_m relative to its MHC-1 bound counterpart.
